# Figuring the ‘cynical scientist’ in British animal science: the politics of invisibility

**DOI:** 10.1057/s41292-023-00312-z

**Published:** 2023-09-26

**Authors:** Tarquin Holmes, Carrie Friese

**Affiliations:** Sociology Department, London School of Economics and Political Science, London, UK

**Keywords:** Animal research, Antivivisection, Scepticism, Cynicism, British nationalism, Invisibility

## Abstract

This paper investigates the ‘cynical scientist’ as a figure in British animal science discourse that developed in relation to the nineteenth-century emergence of the ‘sceptical scientist’. Here, efforts by scientists to demarcate their profession’s territory led to religious backlash against an alleged ‘divorce’ of British science from Christian morality. Animal experimentation became embroiled in this controversy through antivivisectionists’ conviction that animal research was symptomatic of scientific scepticism and Continental atheism’s malign influence. Accusations of cynicism ultimately forced British scientists to accept legal regulation following the 1875 Royal Commission on Vivisection. British scientists were, however, able to utilise their political leverage and credibility as experts to favourably influence licensing and inspection. We suggest that efforts to silence public claims of scientific cynicism may have enabled ‘cynical scientists’ to remain invisible and that this was marked by privilege and power, not marginality. Nevertheless, we argue that regulation and reforms have also worked to internalise within British animal science the notion that scientific cynicism must be combatted through proper governance and internal discipline.

At the close of a conference, attended by Friese, that brought together social scientists and historians of laboratory animals with various stakeholders involved in the contemporary use of animals in scientific research, a veterinarian stood up and asked the question: “How are you going to address the cynical scientists who aren’t at meetings like these?” What brought this conference together was a shared concern with and cares about the welfare of and care for laboratory animals that are used as part of science. Joanna Latimer (2019) has emphasised that gatherings such as these create among participants moments “of affirmation of their capacity as humans to transport themselves into a space outside of the time otherwise constituted ‘as others want them’. Being so gathered shifts them towards ‘being alongside’ (Latimer 2013) in partial and intermittent connection, even perhaps to an intimate entanglement that enjoins them into recovering a world in common” (Latimer 2019, p. 21). The conference did indeed create such a shared sense of affirmation across the sciences, social sciences and humanities. But what this veterinarian was worried about were the scientists who do not attend these kinds of meetings, and who are not concerned enough about the welfare and care of laboratory animals to become “attached” (Latimer 2019) to conferences such as these and to our worldmaking aspirations. Their absence limited the extent to which we could create a common where laboratory animal care is the front and centre of “good science” (Thompson 2013; Druglitrø 2018) as this is presumably not as they want it. The figure of the cynical scientist reminded Friese that we are still marginal, resisting a world that is as others want things to be.

The term ‘cynical scientist’ is useful; Friese had a kind of “ah-ha” moment in hearing it used. With the trope ‘cynical scientist’ this veterinarian crystalised a figure, one who has influenced the design of her research program.^1^ This figure is always present, through their absence. Whilst conducting research, several scientists have told her that the real problem she would face is accessing those scientists who care less about animal care. They are not organised into a “social world” (Clarke 2005; Clarke and Montini 1993; Clarke and Star 2008), actively resisting efforts to embed animal care into science. But they instead quietly resist, by doing the minimum requirements of animal care that are required by law and thus keeping care at the periphery of science.

This paper seeks to understand the figure of the cynical scientist through a genealogical analysis that roots this discursive figuration in that of the sceptical scientist. We understand cynical and sceptical scientists as discursive constructs, but ones that also work to articulate particular ways of doing science. There has long been a motivation for scientists to “detach” (Latimer 2019) themselves from animals and care, even as they are nonetheless deeply attached to a science requiring the use of animal bodies in research. This detachment is generally understood as linked to notions of objectivity, where attachment to the animal is viewed as sentimental and risks introducing unwanted biases. In her ethnography of the everyday moralities at play in laboratory animal science, Lesley Sharp (2019, p. 174) cites Cindy Buckmaster’s description of this as a past ‘Dark Age of Detachment’. In this paper, we seek to understand how and why sceptical and cynical scientists nonetheless persist as discursive figures of concern.

Methodologically, we contribute to a scholarship seeking to understand the lack of presence and sites of (potentially strategic) silence. The invisible and the silent has been an important theme in social studies of laboratory animals specifically (Sharp 2019), and social studies of science and medicine more generally (Clarke 2005; Clarke and Montini 1993; Star 1999; Casper and Moore 2009). In this scholarship invisibility and silence instantiates marginality, wherein power relations render some bodies invisible. We also look at how invisibility and silence instantiate power relations, but rather than bearing the mark of marginality here we explore how the ability to be silent and remain invisible marks privilege and becomes a strategy for maintaining the status quo. But at the same time, invisibility and elusiveness becomes a discursive resource in demands for greater regulation.

This paper is organised as follows. We start by identifying the cynical scientist as related to the sceptical scientist, a figuration through which science and materialism was demarcated from religion and emotion in Britain. This allows us to historicise ideas about objectivity and detachment. We then turn to the nationalistic conditions within which this demarcation was articulated, wherein a particularly ‘British’ kindness to animals was used to assert a hierarchical ranking not only of class in Britain (Ritvo 1987) (and of whiteness in its colonies; see Chakrabarti 2012) but also of nation vis-à-vis Europe. We discuss how ‘divorcing’ knowledge from sympathy was perceived as endangering this hierarchy. We further show how proposals to control cruelty at the 1875 Royal Commission centred around methods of making visible, with an inspectorate ultimately approved in preference to more panoptic propositions which scientists argued would impinge upon and threaten their hard-won social privileges. We discuss how regulation and antivivisectionist agitation drove increasing professional solidarity and internal discipline among scientists, who further utilised their political influence and expert credentials to influence the licensing and inspection system and challenge antivivisectionist attempts to make charges of cynicism publicly visible. We suggest that scientists’ professional consolidation and collective action against claims of cruelty may have created conditions within which cynical scientists could remain invisible to external scrutiny. We conclude by noting that concerns about cynicism have remained persistent in British animal science but that community implementation of the 3Rs and regulatory changes following 1986’s ASPA reforms have opened up spaces for concerns to be raised internally as part of a new governmentality that emphasises the importance of a ‘culture of care’.

Our mode of questioning contributes to better understanding a tension in the social science and humanities scholarship that explores contemporary scientists’ attitudes towards laboratory animals. This literature has often shown that scientists distance themselves from laboratory animals, understanding animals as ‘tools’ rather than sentient creatures (Birke et al. 2007, pp. 11, 14). Whilst animal care work has been professionalised since the middle of the twentieth century (Kirk 2010, 2014, 2008, 2012; Druglitro 2018), it was nonetheless marginalised relative to science per se. This was evidenced by the systematic erasure of animal husbandry practices from scientific journal articles (Lynch 1989; Holmberg 2011; Birke et al. 2007; Lederer 1992). In this context animal husbandry (i.e. the work of feeding, housing, handling and reproducing laboratory animals) was thought an extra-scientific concern that animal technicians and veterinarians, but not scientists themselves, are responsible for (Holmberg 2011; Birke et al. 2007; Greenhough and Roe 2011). Indeed, research indicated that scientists do not see animal care as part of science (Lynch 1989), and notions of objectivity had been used to support this (Birke et al. 2007).

However, social science research has also shown that scientists care quite strongly about animal care (Dam and Svendsen 2017, Online; Svendsen and Koch 2013; Nelson 2018; Davies 2012; Davies 2010; Hobson-West and Davies 2018), not least for the utilitarian reason that poor care creates confounding variables (Asdal 2008). The 3Rs—replace, reduce and refine animals in life science research, first laid out in Russell and Burch’s 1959*Principles of Humane Experimental Technique*—is an important historical moment for understanding this seeming contradiction in social science research.

Importantly, as Tone Druglitrø (2018) has shown, the mid-twentieth century development of the 3Rs was also not an exclusively British phenomenon, with transnational efforts to create international standards for good animal science heavily informing the 3Rs’ conception. Our focus on the contested place of animal experimentation in British society should not suggest we believe this discourse to have been strictly enclosed by national borders, but rather that ideas of ‘Britishness’ and of an associated national culture of caring about animals have played a prominent role historically and today in shaping debates about laboratory animal welfare in Britain, linked to ongoing debates regarding the social value of science and the social standing of scientists. Through our focus on the cynical scientist, we aim to map not only how the apparent contradiction between scientific detachment and care emerged historically but also how concerns oriented around this figure both informed legislation and shaped internal discipline within animal science, and how the cynical scientist as figure of concern complicates the story of the implementation of ethical measures such as the 3Rs through its persistence in spite of reforms.

## Scepticism, cynicism and objectivity in science

The origins of the cynical scientist as a figure of concern in British animal science discourse can be genealogically traced back to earlier Victorian debates surrounding animal experimentation, linked to the public emergence of another figure, the sceptical scientist. These two figures differ in significant ways—a cynic takes a negative view of a claim, potentially despite evidence, whereas a sceptic needs to see ‘valid’ evidence before accepting a claim. But because what counts as ‘valid’ is often contested, one person’s sceptic can be another’s cynic and vice versa. The two figures are thus distinct but sometimes difficult to distinguish (so often conflated). Scepticism and cynicism, we show, were further understood by many Victorian antivivisectionists as related, with scepticism prior to and not necessarily entailing cynicism but cynicism as developing out of and enabled by a sceptical outlook and social atmosphere.

Unlike cynicism, scepticism and detachment have been regarded in moderation as epistemic and scientific virtues, notably by Robert Merton who identified ‘organised scepticism’ and disinterestedness as two integral norms of the scientific ethos and community (Merton 1973). Norms and notions of scepticism and detachment as virtuous, however, have been historically contingent and contested. As Daston and Galison (2007) argue, the notion that scientific objectivity requires setting aside feelings and emotions is a relatively novel idea emergent in the nineteenth century, drawing inspiration from technical innovations in engineering and, particularly, photography.^2^ In the eighteenth century, by contrast, an ideal of ‘truth to nature’ dominated, whereby scientific observation required engagement and attachment to its objects in order to determine underlying forms and types.

Like Daston and Galison’s ‘mechanical objectivity’, we place our sceptical scientist’s origins in the nineteenth century and as manifested in a purported detachment from laboratory animal suffering. We note that this figure is to an extent a postcursor of older figures, such as the seventeenth-century Cartesian rationalist, with their dual doctrines of methodological doubt and beast machine. However, whilst sharing scepticism towards animal pain, Cartesians insisted their beliefs were grounded in clear and distinct ideas, securing foundations for metaphysical truth. The nineteenth-century scientists who articulated scepticism as an epistemological approach were by contrast empiricists who demarcated metaphysics as unknowable, denying the possibility of rational foundations for a priori truths.

## Agnosticism and the demarcation of science and religion

Although we have focussed on ‘cynical scientists’ as figures of concern among contemporary animal welfare advocates, ‘sceptical scientists’ are also sometimes invoked. Allen and Beckoff (2007), discussing debates in ethology, use “skeptical scientist” to denote those opposed to ascribing significant mentality to animals, e.g. “Tail wagging is hardly likely to convince the skeptical scientist”. They partly trace the origins of scientific scepticism in ethology to rejection of Darwin and Romanes’ “anecdotal cognitivism” by C. Lloyd Morgan, who in 1894 coined ‘Morgan’s canon’, the principle that “in no case may we interpret an action as the outcome of the exercise of a higher psychical faculty, if it can be interpreted as the outcome of the exercise of one which stands lower in the psychological scale”.

Morgan’s scepticism regarding animal intelligence claims likely reflected his 1870s training under Thomas Henry Huxley (Peterson 2016, p. 23). A highly influential Victorian scientist and public intellectual, Huxley coined the term ‘agnostic’ in the late 1860s to describe an empirically oriented philosophy of scientific scepticism that held metaphysical truths regarding the underlying nature of reality to be unknowable (Lightman 2002). A particular target of Huxley’s agnosticism was religious dogmatism, maintaining that “scepticism is the highest of duties; blind faith the one unpardonable sin” (Byun 2017). Agnosticism was therefore not only an epistemological theory but also a theological and political challenge to the conservative Anglican British establishment.^3^

Huxley was part of a larger sceptical movement whose ‘agnostic Bible’ was Herbert Spencer’s 1860 *First Principles*. Spencer and Huxley were both influenced by Kant’s critical philosophy, particularly his argument for strict limits to human knowledge, but followed Scottish ‘common sense’ philosopher William Hamilton and Oxford theologian Henry Mansel in rejecting Kant’s claims that practical reason could provide independent grounds for religious and ethical claims. Mansel, *contra* Biblical criticism, advocated for a fideist deference to Scripture based on human reason’s incapacity to comprehend Divine intentions. Huxley and Spencer subverted Mansel by arguing that limits to human knowledge likewise rendered groundless theologians’ pretensions to claim knowledge of metaphysical truths. God could only, in Spencer’s view, be treated as ‘the Unknowable’ (Lightman 1987).

Whilst not antireligious, the agnostics believed ‘dogmatic’ traditional Christian theology was an obstacle to reconciliation between science and religion, each which should be assigned their proper place. The strident public efforts of Spencer and Huxley’s colleague John Tyndall (1874) to demarcate science and religion would prove pivotal in shaping popular images of scientific scepticism in Britain and the Anglophone world (Turner 1978; Gieryn 1999). In his August 1874 Belfast Address to the British Association, Tyndall pondered “the problem of problems”, namely how to satisfy the “immovable basis of the religious sentiment in the nature of man” whilst protecting science from religious supremacism’s “mischievous” forces. Tyndall argued that whilst religion should be barred from “the region of *knowledge*”, where its influence produced “dogmatism, fanaticism, and intolerance”, it was “capable of being guided to noble issues in the region of emotion, which is its proper and elevated sphere”. Knowledge should be the reserve of science, which was ‘empirical’, ‘disinterested’, ‘sceptical’ and able “to abandon all preconceived notions, however cherished, if they be found to contradict the truth” (Gieryn 1999, pp. 50–51).

Like Huxley and Spencer, Tyndall’s target was not general religiosity but rather traditional Christianity, particularly, as a unionist Anglo-Irishman, the Catholic Church. However, his declaration that “All schemes and systems which infringe upon the domain of science must, *in so far as they do this*, submit to its control, and relinquish all thought of controlling it” was widely interpreted as demanding religious deference to science, sparking a widespread backlash (Turner 1978, pp. 373–374). In his 1876 *Problem of Problems*, the American theologian Clark Braden lambasted Tyndall’s Belfast address as “a marauding excursion into the territory of the religious world” (Braden 1876, p. 94). He further denounced “the skeptical scientist” who “ignores the religious and spiritual element of our nature, and utterly discards the plainest utterances and intuitions of the highest” (Ibid: vii). It was this sceptical neglect of the “regnant element” of human nature that Braden and other conservative religious critics of Tyndall targeted. Theirs was a natural theological outlook whereby the role of science was as much to reveal God’s work as to augment human empire. On this view, detaching knowledge production from Christian compassion would detriment the cultivation of religious feeling and induce a drift towards atheism and demoralisation.

The Belfast Address was near-coterminous with the vivisection controversy spurred by Magnan’s experiments on dogs at the British Medical Association’s August 1874 meeting at Norwich. Antivivisectionist concerns, expressed at the following year’s Royal Commission and in subsequent writings, mirrored Tyndall’s critics in believing science to increasingly reject religious and moral authority, so risking a demoralising descent into godless materialism.^4^ “All this iniquitous torture of our weaker fellow-creatures”, warned George R. Jesse, “tends to the incarnation of evil, namely, intellect divorced from moral principle” (1875, p. 127). George Duckett similarly saw vivisection as “horrid and monstrous” and “hand in hand with Atheism” (Report 1876, p. 326). James John Garth Wilkinson regarded vivisection and “sceptical science or materialism” as explicitly linked (1876, p. 198), condemning the “vehement antitheological bent of science at present” and diagnosing “its atheism” as “coincident with its horrible cruelty at Norwich” (Ibid: 111–112). Edward Maitland likewise lamented “the divorce now subsisting between knowledge and sympathy”, with vivisection threatening “their everlasting estrangement” (Maitland 1913, p. 83).

Whilst many attacks on sceptical science focussed on its perceived antitheological pretensions, its advocacy of detachment and doubting empiricism was also criticised, antivivisectionist doyenne Frances Power Cobbe maintaining that the real issue with sceptical science was not its iconoclastic character but its nihilistic indifference. “It is contented”, she remarked, “to be Agnostic, not Atheistic. It says aloud, ‘I don’t know’. It mutters to those who listen, ‘I don’t care’” (Cobbe 1888, p. 34).^5^

## Science, religion and animals in the formation of British nationalism

Nineteenth-century antivivisectionists’ concerns about a ‘divorce’ between knowledge and sympathy can be understood as linked to ideas of a peculiarly British scientific style and ethic, reflective and respective of the belief in a shared national sentiment of kindness to animals and perceived as threatened by atheistic and callous Continental materialism. In its concerns about laboratory animal treatment, British antivivisectionism also articulated xenophobic fears that European science’s influence would uncouple the British scientific community from the nation’s moral sentiments. Continental science was thought a threat, we will illustrate, because of the ways which British attitudes towards animals and science were intertwined with British nationalist sentiments.

The English in the early modern period were “notorious among travellers for their cruelty to brutes” (Thomas 1984, p. 144), and as late as the early nineteenth century “would have been surprised to hear themselves praised for special kindness to animals” (Ritvo 1987, pp. 125–126). Advocacy for humane animal treatment, however, began rising in the half-century before 1800 (Tague 2015). This period also saw Britain’s consolidation as a political and cultural unit amid near continuous war with European rivals, particularly France. ‘Britishness’ emerged as a unifying identity marked originally by shared ruling Protestant class commitment to opposing Catholic continental supremacy (Colley 1992). Early animal advocates indeed often employed anti-Catholic arguments, e.g. citing the Vatican’s position that animals did not have souls (Thomas 1984, p. 144).

The onset of the French Revolution and Napoleonic Wars produced new British ruling class anxieties, articulated in new forms of xenophobia. Edmund Burke influentially argued that revolutionary republicanism’s disdain for tradition and social hierarchy would lead to widespread social demoralisation, with utilitarian justifications of political violence, by “present[ing] a shorter cut to the object than through the highway of the moral virtues”, leading ultimately to “insatiable appetites” for “rapacity, malice, revenge” (Burke 1910, p. 79). Ritvo (1987) advances that late eighteenth-century ruling class concerns about demoralisation and social unrest informed a new large-scale animal advocacy movement that pressed for formal legislation against cruelty and to control ‘dangerous classes’. This culminated in the first national anti-cruelty legislation, the 1822 Cruel Treatment of Cattle Act, and in the RSPCA’s 1824 foundation. These advocates synthesised Burke’s belief in revolutionary upheaval’s demoralising influence with older concerns that those who harmed animals would go on to harm humans, e.g. as depicted in Hogarth’s 1751 *Four Stages of Cruelty*. The early nineteenth century thus saw the constitution of a new national order oriented around controlling the lower classes through regulating their treatment of animals. This led by the mid-nineteenth century to bans on most working-class bloodsports and increased surveillance and regulation of worksites associated with animal cruelty, e.g. slaughterhouses and omnibus routes. Ideologically, kindness to animals became linked to efforts to preserve social order rooted in British class hierarchy.

Whilst the rationale linking animal abuse to demoralisation justified increasingly intrusive scrutiny of working-class relations with animals, it also made it imperative that members of the British ruling classes^6^ performatively distance themselves from cruel practices. Vivisection early on troubled efforts to perform such distancing. In February 1825, MP Richard Martin, architect of the 1822 Cattle Act, condemned French physiologist François Magendie as a “disgrace to society”, following allegations he had vivisected “a ladies’ spaniel” before a crowd of London doctors and scientists during his previous year’s visit (Hansard 1825, pp. 1011–1012; Olmsted 1944, pp. 134–143). Martin’s denunciation placed practitioners of vivisection among Britain’s medical elite in a difficult position, given the threat posed by accusations of demoralisation to their status as members of the upper and educated classes. British physiologists, led by Charles Bell, responded, arguing they differed from Magendie and his French counterparts in basing their claims on detailed anatomical study, only deploying vivisection, if at all, to confirm theories. This claim did reflect some methodological differences between contemporary British and French physiology (see Berkowitz 2015, pp. 145–146). However, Bell, in condemning French physiology as cruel and sloppy empiricism, elided that his claimed proof of the differential functions of the anterior and posterior nerve roots (focus of an ugly priority dispute with Magendie), was attained through vivisecting a donkey’s spinal nerves (Olmsted 1944, pp. 103–104; Berkowitz 2015, pp. 139–140).

By claiming British science to scorn vivisection, Bell both defended British physiologists against accusations of demoralisation and also contributed to the national myth of the British as animal lovers. In maintaining that British scientists participated in broader British society’s sentiments, Bell helped moreover to diffuse tensions within the ruling class between science and animal advocates. Bell further contributed to linking British science and society through his natural theological writings, in 1833 authoring the fourth Bridgewater Treatise, ‘The hand’ (Amundson 2005, pp. 64–65). British natural theology had seen an early nineteenth-century revival in a reaction against materialism, associated with atheism and revolutionary France.^7^ It offered a complementarian marriage of science and morality, whereby “the pursuit of science was an act of piety, and every discovery furnished additional evidence of the wisdom and goodness of God” (French 1975, p. 352). Bell was thus influential in shaping a view that British science, informed by animal-loving sentiment and Protestant piety, differed from European, particularly French, physiology, alleged to participate in Catholic disdain for animals and revolutionary irreligious cynicism.^8^

## Class and the decline of Bellian complementarism

By the mid-nineteenth century, British natural theology’s influence was in sharp decline. New secular theories of geological and biological development such Lyell’s uniformitarianism and Darwin’s natural selection challenged religious arguments from design. The professionalisation of British science, linked to the expanding industrial and imperial economies’ demand for trained expertise, also marginalised previously prominent parson naturalists (Turner 1978, pp. 364–367). Medicine likewise underwent professionalisation (French 1975, p. 290). The decline of amateurs and churchmen in medicine and science paralleled the political decline of the conservative Anglican aristocratic elites who had dominated British politics since the Napoleonic Wars. Elements of this class conflict influenced antivivisectionist depictions of scientific scepticism and physiological cruelty. Cobbe complained that most British doctors were now “sons of men of the secondary professional classes or of tradesmen”, and were thus “a parvenu profession, with all the merits and the defects of the class”, i.e. unrefined and lacking true gentlemanly character (French 1975, p. 341). Drawing parallels between physiologists and revolutionary French republicans, she further declared science as now “essentially Jacobin” (1888, p. 27), alleging vivisectors to share a “greediness of sight of horrors” with “the women who sat gloating as they watched the guillotine at work in the old French Revolution” (2004, p. 287). Echoes of Cobbe’s classism and Burke’s 1790s arguments against revolutionary zeal can also be found in Rev. John Verschoyle’s claim that “It is an axiom, apparently, with the supporters of vivisection… that by the path of immediate expediency the goal of human progress is to be reached”. He viewed this “inclination to take the short-cut to any desired goal” as an “inclination of common [i.e., lower class] minds” (Verschoyle 1884, p. 232).

Supporters of physiology likewise regarded antivivisection as threatening the elevated social prestige that for many doctors and scientists was only recently hard-won. At the Royal Commission, Chief Medical Officer John Simon rejected legislation precisely on the grounds of treating physiologists as “a dangerous class” that should be “licensed and regulated like publicans and prostitutes” (Report 1876, p. 75). Against accusations of ‘blunted feeling’ and bad character, physiologists made great efforts to emphasise their sensitivity to human and animal suffering, appealing to classist assumptions about the alleged greater sympathetic capacities of the British higher orders (for more extensive discussions see Boddice 2016; Holmes 2021).

Tyndall’s Belfast Address and the Norwich prosecutions were thus preceded by several decades’ slow decline of Bell’s attempted marriage of British science to animal-loving sentiment and theology to deflect accusations of cynical cruelty linked to vivisection. ‘Divorce’ was felt by many British conservatives as “something very much on the order of a betrayal” (French 1975, p. 363), Belfast and Norwich fuelling calls for “remarriage” (Maitland 1913). This outrage had strong nationalistic, religious and class dimensions, associating vivisection with foreigners, atheists and parvenus and seeking a restoration of traditional complementarian values in science and medicine. In turn, scientists and medical men saw antivivisection, in portraying physiologists as a ‘dangerous class’, as threatening their professional prestige and political advances. It was therefore imperative to defend sceptical science against antivivisectionist accusations of its cynical potential.

## The 1875 Royal Commission—the role of the sceptical scientist in the collapse of belief in British scientific exceptionalism

At the 1875 Royal Commission on Vivisection, some scientists continued to maintain that, whilst cruelty might be rampant abroad, British science and society were different. Bell’s former protégé John Anthony declared the ‘English system’ “infinitely superior” to that of the French, whom he declared “very careless of what becomes of the animal”. Asked why he thought the French crueller, he responded “I think that nationality has something to do with it” (Report 1876, pp. 130–137). Francis Sibson similarly defended the right of ‘English physiologists’ to make the same experiments as performed in France and Germany on the basis that “they may set continental nations an example how to produce the same results in a scientific point of view with greater humanity. That has always been the aim of the English experimenter” (Ibid: 237). George Henry Lewes concurred that the ‘English’ were kinder, maintaining there to be no need for controlling legislation as, unlike continental Europeans,

our morality has certainly been cultivated in the direction of greater sympathy with animals; and of course the surgeons and medical men are taken out of the general mass of the population, and in those countries of which I have spoken they bring with them what I should call the national indifference. In England we are not indifferent to those things; at least not so much (Ibid: 312).

Cracks, however, appeared in this consensus. Edinburgh’s William Rutherford, who trained at Berlin and Leipzig, refused to countenance that Continental vivisection was crueller, maintaining that the practices he witnessed in Europe were “exceedingly humane” (Ibid: 152). Privy Council medical officer John Simon likewise refused, when asked, to speculate whether Italian practices were more careless, and went so far as to criticise the Bellian myth that British scientists scorned vivisection, pointing out that Bell’s own student, Herbert Mayo, conducted “severely painful operations” (Ibid: 75).

Much has been written on Emanuel Klein’s explosive testimony, especially his notorious declaration of having “no regard” for animal suffering and only using anaesthetics to keep his charges quiet (Ibid: 183). French (1975, p. 103) argues that Klein’s testimony altered the Commission’s course in forcing Huxley, acting Commissioner for the ‘scientific interest’, to agree to the need for meaningful legislation. Indeed, Huxley privately declared to Darwin that he regarded Klein “an unmitigated cynical brute” and would “willingly agree to any law, which should send him to the treadmill” (Huxley 1908, pp. 171–172). Klein’s further profession that a good experimentalist must pay little attention to the animal’s feelings and instead direct their “whole attention” on “making the experiment, how to do it quickly, and to learn the most that he can from it” (Report 1876, p. 183) certainly also influenced public perceptions of scientific scepticism and belief in attendant risks of cynical cruelty.

Klein, however, was a Vienna-trained Austrian Jew, not a British national. His statements on experimentation moreover differed little in tone from those of Magendie’s successor Claude Bernard, who held that the physiologist should be “a man possessed and absorbed by a scientific idea”, deaf to “the animals' cries of pain” and “blind to the blood that flows”, seeing only “his idea, and organisms which conceal from him the secrets he is resolved to discover” (Bernard 1949, p. 103). Given his background and Bernardian convictions, why could not Klein simply be dismissed as another case of continental callousness?^9^

Klein himself did not think British scientists much different from their continental colleagues. Whilst agreeing that ‘English’ people and Europeans differed in sentiments towards animals, he did not believe British physiologists to partake in their compatriots’ sympathies (Report 1876, p. 183). This concurred with the suspicions of many antivivisectionists, who had already identified several British-born physiologists’ practices as cruel, including Rutherford (use of curare instead of proven anaesthetics) and David Ferrier (cerebral localisation experiments on animal brains). Ferrier’s counterclaim to treat animals humanely was undercut by RSPCA Secretary John Colam’s testifying that Ferrier mocked his experimental subjects at a public demonstration, stating “I have heard him say that the animals ‘appeared’ to be in intense suffering, and then joke about the stupidity of the animal, especially if the animal happened to be a monkey” (Ibid: 82). More generally, whilst there was considerable variation among scientific witnesses at the Commission on the nature of animal sensitivity, many of them expressed scepticism regarding claims that human and animal pain were comparable in intensity.^10^ Huxley as the Committee representative ‘for science’ supported these arguments, notably intervening to argue in favour of John Anthony’s claim that “animals of the lower range do not feel pain as we feel pain” (for detailed discussions see Holmes 2021; Hornsby 2019: 105–112).

Although Klein offered a convenient case study of professed scientific callousness, the perceived cynicism of Rutherford and Ferrier, among others, made it difficult to treat physiological cruelty as a problem brought in by outsiders.^11^ This cynicism further appeared to receive cover from a wider scientific scepticism regarding animal sensitivity to pain. Antivivisectionists interpreted this scepticism as evidence of widespread “blunted feelings” among physiologists (Report 1876, p. 211). Scientific scepticism was thus presented as not only antagonistic to religious tradition but as also endangering a humanitarian national ethos’ cultivation, calling into question physiology’s compatibility with ‘British values’.

## Making the cynical scientist visible

If cruelty was not simply brought in from outside, this suggested cruelty might be hidden within the British scientific community. To restore public trust in British science required creating means to make cruelty visible. In a previous paper (Holmes and Friese 2020), we discussed concerns at the 1875 Commission about the shelter from scrutiny afforded to physiologists by laboratory walls. The antivivisectionist doctor George Hoggan thereby suggested that not only should all private vivisection be banned (a view many witnesses at the Commission concurred with), but more radically that all physiological experimentation be restricted to laboratories with a built-in gallery allowing public access to and surveillance of scientists (Report 1876, p. 178). Although Hoggan’s proposals were roundly rejected, the system of inspection and licensing instituted by the 1876 Cruelty to Animals Act, in enforcing regular supervision of experiments and mandating physiologists to detail and justify expected experimental pain, can similarly be interpreted as a means to make cruelty more visible. In practice, the inspectorate’s efficiency was initially limited due to its tiny size (three inspectors covering the entire country) plus the need to be on friendly terms with scientists to gain access to labs (Shmuely 2017, pp. 158–222). The Act’s enforcement therefore relied heavily on licensing, which depended on scientists’ self-reporting of their experimental methods.

Antivivisectionists, however, lacked faith in physiologists’ honesty. To cite one critic at length:

How is the painlessness of the experiment to be secured? Of course no one would for a moment think of trusting the physiologists themselves. The eagerness, the heat, the fury of the thirst for knowledge in this department of science, renders them that are engaged in it absolutely unregardful of any pain inflicted in the search, which they have not themselves to bear. Dr. Emanuel Klein is no solitary specimen of the order to which he belongs. He is only a very outspoken one (Wright 1878, p. 10).

Whilst Wright’s characterisation of the vivisector’s psychology was doubtless more born of fearful fancy than empirical evidence, he was nevertheless correct in believing that most British scientists were sensible of public sentiment and made strident efforts to avoid visible appearance, warranted or otherwise, of cynicism towards experimental animals. Antivivisectionists commonly responded to this invisibility by treating all physiologists as suspects.

Meanwhile, physiologists were increasingly encouraged to self-censor references to animal pain to avoid giving grist to antivivisectionist mills. Critics of vivisection within the physiological and medical communities were subjected to ostracisation, to the effect that antivivisection changed rapidly from an accepted if contested viewpoint c. 1875 into a *verboten* conviction that if openly professed was “tantamount to professional suicide” (Bates 2017, p. 152).^12^ This internal discipline was co-ordinated by advocacy groups such as the Association for the Advancement of Medical Research (AAMR), formed in 1882, and its successor the Research Defence Society (founded 1908), who also acted as interlocutors with the Home Office and Inspectorate and advised on licensing (French 1975). Whilst many physiologists doubtless shared Huxley’s disgust with ‘cynical brutes’ like Klein, a hostile and polarised post-1876 atmosphere encouraging self-censorship and public silence about ethical concerns likely cemented the idea that ‘cynical scientists’ remained and yet were invisible.

Physiologists’ efforts to control their work’s public visibility and the ways in which this contributed to the appearance of cynicism extended to using political influence and legal machinations to try to silence antivivisectionists, as seen in the famous Brown Dog controversy. When Louise Lind af Hageby and Leisa Schartau alleged in 1903’s *Shambles of Science* that they had seen a dog with an unhealed wound vivisected by UCL’s William Bayliss (contravening the 1876 Act’s requirement that vivisected animals be euthanised whilst under anaesthesia), it was their inclusion of the chapter ‘Fun’ that raised physiologists’ ire. It claimed, contradicting physiologists’ claims to humanity, that Bayliss and his students joked and laughed throughout the lecture, Bayliss even “comfortably smoking his pipe” with blood-stained hands mid-experiment (Kean 1998, pp. 141–142). This attack on character, suggesting Bayliss was a cynical scientist, led to a libel trial that saw the chapter ordered suppressed as denigrative of Bayliss’ character (Lansbury 1985, pp. 11–12).

Lind af Hageby and her supporters’ response sought to firmly push vivisection back into the public eye, in 1906 unveiling a statue commemorating the dog with an inscription alleging the animal had “endured Vivisection extending over more than Two Months” and been “handed from one Vivisector to another”. This outraged UCL medical students, who rioted and attempted to demolish the statue before its eventual surreptitious destruction by Battersea Council in 1910 (Ibid: 13–22). Overall, it is notable how much controversy and violence surrounding the Brown Dog case centred around the visibility of accusations of scientific cynicism and cruelty.^13^ The AAMR’s Stephen Paget went as far as suggesting the statue could remain but the inscription be altered to state that the dog had been “free of all pain” and “just died in its sleep” (Ibid: 20). It was not the publicisation of the fact of vivisection that angered scientists but rather the claim of cynicism.

## The persistence of concerns

Concerns about ‘sceptical’ and ‘cynical’ scientists appear to have receded in Britain with the beginning of WWI. Bates observes (2017, pp. 141–142) that public concern was redirected elsewhere in this context, and many antivivisectionist societies suspended their activities. Other factors, however, also contributed to the marginalisation of the controversy, not least the increasing proof of medical advances developed through animal experimentation (e.g. diphtheria antitoxin), social goods that rendered concerned publics more accepting of vivisection. Meanwhile, there was also increasing recognition among scientists of a need for developing more holistic approaches to animal welfare. Kirk (2014) has documented how in the interwar and early postwar period ethological studies greatly increased understanding of the multiple psychological and psychosomatic causes of laboratory animal suffering and their influence on experimental outcomes.

Despite these changes in scientific attitudes to welfare, public concerns about scientific cynicism persisted across the twentieth century, sustained by periodic outbreaks of controversy when scientific practices were decried as out of step with British public sentiments. New animal welfare movements in the 1960s and 1970s further articulated old concerns about the dangers of detachment from animals together with novel calls for reform to farm animal welfare and recognition of animal rights (for continuities between Victorian/Edwardian and postwar British animal activism, see Kirchhelle 2021). The figure of the cynical scientist thus persisted in the public imagination through the course of societal and scientific change.

Concerns about cynicism also originate from within science itself, as seen in the opening ethnographic vignette and in the historical material. Whilst French (1975) suggests that the influence of groups like the AAMR and RDS over licensing ultimately helped rendered the 1876 Act relatively toothless, Shmuely (2017) has against this argued that the AAMR ultimately came to collaborate with the Inspectorate to help ensure that experimental design and operation remained strictly within the law’s limits. In this way, liaisons between leading scientists and inspectors helped impose internal discipline among animal scientists to help counter perceptions that scientific culture diverged from national culture.

Russell’s development of the 3Rs in the 1950s reflected a continuing concern with internal discipline and can likewise be seen as an attempt to impose governmentality within British animal science through the 3Rs. Specifically, Russell sought to “diminish inhumanity in experimentation” (Russell and Burch 1959) by cultivating a scientist subject who is humane to animals (Kirk 2018). Russell maintained that the main sources of inhumanity in the laboratory were insufficient education and ‘authoritarian’ personalities and advocated countering these with a combination of training in humane experimental technique and acculturation within a humanitarian group ethos.

Importantly, however, Russell left open the question of how to manage already institutionally embedded scientists who reject and passively resist humane method and acculturation, i.e. those identified as ‘cynical scientists’ by animal welfare proponents such as the veterinarian cited in the opening vignette. This question of how to manage expert indifference and cynicism towards laboratory animal welfare, especially among higher ranking scientists, troubled discussions on how to institute Russell’s recommendations. That the 1986 Animal (Scientific Procedures) Act, in addition to laying the groundwork for the eventual mandating of the 3Rs, also empowered animal care workers to better hold license holders to account through establishing statutory officerships for veterinarians (NVSs) and senior animal technicians (NACWOs) (Kirk and Myelnikov 2022) can be seen as an admission that implementing the 3Rs required not only a humane laboratory culture but also a more democratic work environment in which the voice of perceived ‘animal advocates’ held weight. And yet, despite these reforms, there continue to be cases where cynicism seems to become embodied, most recently with the 2013 Imperial College scandal. The figure of the cynical scientist therefore persists as a personified stereotype of scientists who resist the efforts from both within science and beyond to make animal care part and parcel of “good science” (Thompson 2013; Druglitrø 2018).

These insights gained from our focus on the cynical scientist contribute to a recognition, as made clear in a recent special issue (Davies et al. 2018), that the story of the implementation of the 3Rs is less straightforward than often presented, with clear breaks and ruptures between *The Principles*’ commissioning by the Universities Federation for Animal Welfare (UFAW), its 1986 implementation in the UK’s Animals (Scientific Procedures) Act (ASPA), and the ways in which the 3Rs are now positioned as the—albeit contested (McLeod and Hartley 2018)—transnational gold standard in laboratory animal welfare and ethics. That concerns about sceptical and cynical scientists persist further suggests that we should understand implementation as a still ongoing process entailing structural as well as cultural reform, and which may never be truly ‘complete’.

## Conclusion: the politics of invisibility

The existing literature on the sociological salience of invisibility and sites of silence, largely developed in US contexts, tends to present invisible actors as marginalised. Women users of RU486 are marginalised and therefore cannot articulate their own positions on the drug but are instead represented others (Clarke and Montini 1993); dominant discourses on infrastructures need to be challenged by seeking out unheard and hence marginalised voices (Star 1999); not being counted in US public health translates into one’s health and life ‘not counting’ (Casper and Moore 2009). In the context of laboratory animals, Sharp identifies three key invisible actors through her ethnographic fieldwork: (1) the laboratory animal; (2) the animal technician and (3) the scientist who opts out of animal research. The first two invisible actors are invisible precisely because of their precarious position in a hierarchical relationship, where animals are generic substitutes for humans and technicians provide a service to scientists. Invisibility is thus a marker of marginality.

The cynical scientist, by contrast, is not a marginal actor, but rather (as the stereotype goes) one who utilises strategies of invisibility, silence and detachment, to maintain the status quo. As a figure, the cynical scientist has long been a source of concern precisely because that figuration may exist in the form of an actor, one who works within the law by performing tick box exercises but does not follow the spirit of the law, seeing regulation as an obstacle to efficiency rather than as mandating a minimal basis for animal welfare. The culture of secrecy and silence linked to fear of extremist violence and concern for keeping oneself and one’s family physically safe provides further cover for the cynical scientist by rendering discussion of what goes on in the laboratory outside of the lab both dangerous and a breach of protocol. Invisibility becomes a marker of relative privilege in this context.^14^

Methodologically, this paper has sought to show how figurations like the ‘cynical scientist’ develop over time, accruing certain meanings that are located spatially and historically. The cynical scientist is not an ideal in the way that the sceptical scientist is, but rather articulates the anxieties that this norm produced. These anxieties shape regulation, both formally in law and informally through cultivating dispositions and organisational practices. In this way, an actor’s category like ‘cynical scientists’ needs to be unpicked as a local articulation that conveys the worries and concerns of normative and transnational scientific aspirations. Britain is here an analytic unit for conducting this type of analysis.

## Footnotes

1 For a discussion of studying figures methodologically, see Moser and Schlechtriemen (2019).

2 Tyndall, arguing for the virtues of detachment and sceptical empiricism, notably employed the metaphor of the scientific mind as photographic plate (Gieryn 1999, p. 48).

3 Although one which, in its unusual interpretation of Kant and strident efforts to distinguish itself from ‘Continental’ atheism and materialism, might be considered peculiarly British.

4 Tyndall, like Huxley, was broadly supportive of animal experimentation and was a champion of Emanuel Klein’s bacteriological research at the Brown Institute (Worboys 2000). This may have further contributed to the linking of Belfast and Norwich in antivivisectionist minds.

5 Cobbe appreciated that most British ‘sceptical scientists’ did not profess atheism, indeed stridently denied scepticism’s affiliation with it, as Tyndall did in 1870 when he argued that evolutionists “have as little fellowship with the atheist who says there is no God, as with the theist who professes to know the mind of God” (Lightman 1987, p. 17). The dangers of agnosticism for Cobbe, that linked scepticism to cynicism, were therefore not outright denial of higher moral truths but indifference to their ultimate nature.

6 Nineteenth-century British debates around animals often ostensibly centred around ‘English’ values and ‘Englishness’. At the 1875 Royal Commission, antivivisectionists appealed to the “common sense of the English nation” (Jesse 1875, p. 106), whilst Oxford anatomist Henry Wentworth Acland declared the “scientific sense of England” as called to decide on matters (Report 1876, p. 42). However, as Reuben Message observed in conversation with us, many prominent critics of animal cruelty and vivisection—including Richard Martin and Frances Power Cobbe (Anglo-Irish), and Lord Erskine and Charles Bell (Scottish)—were actually from the Celtic periphery. The cultivation of a national ethos of kindness to animals should thus be seen as the work of a *British* (not merely English) elite, and as helping constitute a novel British identity emergent out of the eighteenth- and nineteenth-century integration of England, Scotland and Ireland into a unitary state. Further, ‘English’ and ‘British’ were often in this period used interchangeably, reflecting a certain fluidity in cultural identification. Bell, despite his Scottish origins, was for example described at the Royal Commission as an “English” physiologist and experimenter (Ibid: 132, 237).

7 Darwin’s grandfather Erasmus was for example attacked for his proto-evolutionary work *Zoonomia* (Desmond and Moore 2009, pp. 3–4).

8 Anti-Catholicism remained popular among late nineteenth-century antivivisectionists, e.g. Methodist Rev. H.J. Piggott (1882, pp. 529–530) citing the “atmosphere of cynical scepticism” in Catholic Continental Europe as enabling French and German vivisection.

9 Antivivisectionist responses to the Commission remained marked by xenophobia, Victoria Street expressing continuing concern at the “importation of foreign teachers” and “influence of foreign example in English physiological laboratories”, particularly “the principles and tone of feeling of foreign physiologists” (SPALV 1876, pp. 66–67).

10 Lewes and Joseph Lister both went so far as to question whether frogs actually consciously experienced pain (Royal Commission: 217, 313; see Holmes 2021, p. 205, n. 38 for further discussion).

11 On increasing antivivisectionist hostility to British physiologists’ claims to exceptional morality, see the *Home Chronicler* article ‘Claude Bernard’s Successor’ (Anon 1878, p. 313) regarding the Collège de France’s appointment of Mauritius-born British citizen Charles-Édouard Brown-Séquard: “We were told… that it was the foreigners who were the ‘black sheep’; that the British physiologist… was a most harmless creature, and that we in this country were completely unpractised in the ‘art’ of Vivisection. Now, it turns out that the French have come to England for a successor to the Arch-Vivisector of his day”.

12 Bates cites the case of BUAV president Walter Hadwen, who was “barred from joining the BMA, and was subjected to what appears to have been a vexatious trial for medical manslaughter after the death of a patient in 1924” (2017, p. 152).

13 Compare with Kuchinskaya’s (2014, Ch.3) analysis of the politics of (in)visibility (linked to conflicts between government and political opposition) surrounding the Chernobyl disaster in 1990s Belarus.

14 Invisibility and the reproduction of privilege is also a theme in critical whiteness studies (see, for example, Sue 2006).
